# Neurobehavioral Assessment of Sensorimotor Function in Autism Using Smartphone Technology

**DOI:** 10.1002/aur.70166

**Published:** 2026-01-03

**Authors:** Kayleigh D. Gultig, Cornelis P. Boele, Lotte E. M. Roggeveen, Ting Fang Soong, Seth Sherry, Caroline Jung, Sara Milosevska, Anton Uvarov, Khalid Benhassan, Said Ait BenAli, Yasmine Ahajoui, Valeria Carpio‐Arias, Sander Lindeman, Sebastiaan K. E. Koekkoek, Esra Sefik, Myrthe J. Ottenhoff, Samuel S.‐H. Wang, Chris I. De Zeeuw, Abdeslem El Idrissi, Henk‐Jan Boele

**Affiliations:** ^1^ Department of Neuroscience Erasmus MC Rotterdam the Netherlands; ^2^ BlinkLab Limited Perth Australia; ^3^ Neuroscience Institute Princeton University Princeton New Jersey USA; ^4^ Mohammed VI National Center for the Disabled Salé Morocco; ^5^ Department of Neurosurgery Cadi Ayyad University, Mohammed VI University Hospital Center Morocco; ^6^ Department of Psychology FLSHM—Hassan II University Casablanca Morocco; ^7^ Escuela Superior Politécnica de Chimborazo Facultad de Salud Pública Riobamba Ecuador; ^8^ Netherlands Institute for Neuroscience Royal Academy of Arts and Sciences Amsterdam the Netherlands; ^9^ Center for Developmental Neuroscience City University of New York, College of Staten Island New York USA

**Keywords:** autism spectrum disorder, neurodevelopmental disorders, neurophysiological tests, perception, reflex startle

## Abstract

Differences in sensorimotor processing represent an important, yet underrecognized, feature of autism; typically assessed through subjective observations, which, although important, are susceptible to biases. To complement these observations, a more objective approach to assess sensorimotor function may be possible through reflex‐based neurobehavioral evaluations. The clinical application of these assessments has, however, been largely confined to laboratory settings. Thus, small sample sizes and inconsistent findings have made it challenging to understand how sensorimotor function differs in autism and whether it can be used as an objective biomarker for diagnostics. Here we present a novel smartphone‐based platform to conduct neurobehavioral evaluations by measuring facial and behavioral responses in at‐home environments. Through a multi‐center study, we explored the platform's ability to distinguish between children with and without autism. We enrolled 536 children aged 3–12 years. BlinkLab smartphone‐based assessments were successfully completed in 431 children (80.4%), including 275 with autism and 156 neurotypical children. We found that autistic children showed altered sensorimotor responses across multiple domains. These included reduced prepulse inhibition (PPI), stronger startle habituation over the course of a PPI test, more variable eyeblink responses to auditory stimuli and significant sensitization. Additionally, children with autism displayed more screen avoidance, postural instability, head movements, mouth openings, non‐syllabic vocalizations, horizontal pupil shifts, “side‐eyeing”, and variation in baseline eyelid opening. Exploratory analyses showed that these effects were largely independent of co‐occurring conditions. Notably, co‐occurrence did influence certain subdomains (e.g., PPI, mouth openings). These findings illustrate that smartphone‐based assessments can capture distinct sensorimotor profiles associated with autism in real‐world environments.

## Lay Summary

– The way the brain processes sensory information to guide behavior may differ in children with autism.
– In this study, we measured sensory processing in autism by looking at facial and postural reactions to ultra‐short sounds while children watched a video, all done on a smartphone.
– Autistic children were more startled by these sounds and showed distinct behaviors like mouth openings and vocal sounds that were not commonly seen in neurotypical children.
– These findings suggest that differences in sensorimotor processing can be measured with a smartphone application in everyday settings. In the future, these measurements may help us develop new diagnostic tools for autism.

## 1 Introduction

Atypical sensorimotor functioning is highly prevalent in autism (Kanner 1943; Kern et al. 2006; Marco et al. 2011; Mosconi and Sweeney 2015), however, limited attention has been given to sensorimotor skills in both autism research and clinical practice (Coll et al. 2020; Mosconi and Sweeney 2015; Narzisi et al. 2025). Sensorimotor processing is well‐studied in animal and human‐lesion studies (Mosconi and Sweeney 2015), making it a powerful research tool to explore potential neurophysiological pathways underlying behaviors observed in autism. Importantly, sensorimotor skills are strongly associated with the level of functioning in autism (Hannant et al. 2016; Mosconi and Sweeney 2015; Patterson et al. 2022; Travers et al. 2017), more specifically with cognitive abilities (Denisova and Wolpert 2024), and may even underlie other, more commonly addressed features of autism including social challenges (Marco et al. 2011; Narzisi et al. 2025). Thus, understanding sensorimotor processing is important from a clinical perspective where objective biomarkers identifiable in early life are important for diagnostic purposes.

Advancements in digital technology have made the quantification of such sensorimotor skills possible through techniques such as computer vision analysis. These quantifications could complement existing established tools in the autism diagnostic procedure, including structured interviews and questionnaires, especially since sensory sensitivity is often harder for a clinician to assess than, for instance, social functioning. Objective biomarkers for autism could potentially contribute to more efficient autism diagnostic pathways (Kanne and Bishop 2021).

An interesting potential biomarker that has not yet been studied with computer vision analysis includes neurobehavioral evaluations assessing sensorimotor function that rely on brain reflexes. Examples include the acoustically evoked eyelid startle reflex (ASR), which can be modified in a prepulse inhibition paradigm (PPI) to measure sensorimotor gating (Braff et al. 2001; Swerdlow et al. 1999) or in the startle habituation paradigm (HAB) to measure non‐associative learning (López‐Schier 2019; Simons‐Weidenmaier et al. 2006). In PPI, the response to a normally startling stimulus is reduced by a preceding weaker stimulus (Braff et al. 2001; Swerdlow et al. 1999). In HAB, a decreased response in startle amplitude occurs with repeated stimulus presentations (Pilz and Schnitzler 1996). Together, ASR, PPI, and HAB can be used to understand different aspects of sensory information processing (Abel et al. 1998; Braff et al. 2001; Graham 1975; Koch 1999; Madsen et al. 2014; Perry et al. 2007; Swerdlow et al. 1999). Differences in these three measures have been found between autism and neurotypical development in pre‐clinical autism research in animal models (El‐Cheikh Mohamad et al. 2023) as well as human studies (Cheng et al. 2018; Kohl et al. 2014; Madsen et al. 2014; McAlonan et al. 2002; Perry et al. 2007); however, these findings are not consistent (Cheng et al. 2018; Oranje et al. 2013; Ornitz et al. 1993; Takahashi et al. 2016, 2017; Yuhas et al. 2011), likely due to small sample sizes, variability in stimulus intensities, and participant characteristics (Cheng et al. 2018; Doornaert et al. 2024).

Another type of biomarker could be more general sensorimotor behaviors also observable in neurobehavioral assessments and easily quantifiable with computer vision analysis. Early identification of autism is important for optimal clinical outcomes (Franz et al. 2022; Zwaigenbaum et al. 2015) and while differences in motor development are not described as a core feature of autism in the DSM‐V, they are reported to be visible in earlier stages of development (Patterson et al. 2022; West 2019; Wilson et al. 2021) compared to social and language development. Differences in motor and sensorimotor behavior are supported by a substantial body of recent evidence. For example, differences in head‐and‐body movements (Campbell et al. 2019; Dawson et al. 2018; Krishnappa Babu et al. 2023; Martin et al. 2018; Zhao et al. 2021, 2022) as well as vocalizations (Tenenbaum et al. 2020) have been shown in children with autism compared to neurotypical children.

**FIGURE 1:**
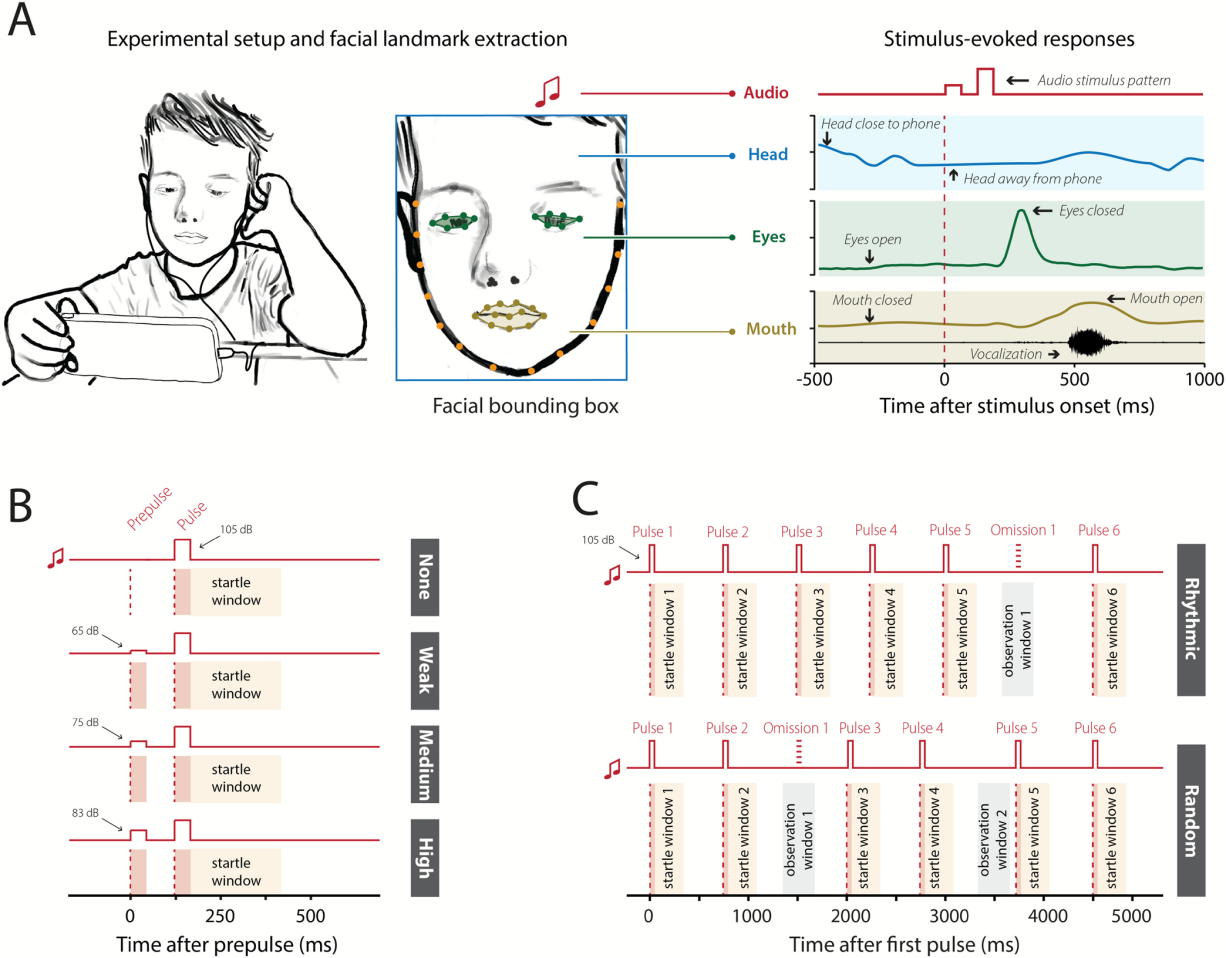
Data were collected using a smartphone‐based platform for neurobehavioral evaluations. (A) Children watched a 15‐min video on a smartphone during which brief auditory stimulus patterns were delivered. The smartphone's camera captured the child's postural, head, facial, and vocal responses before and after stimulus onset. Illustrations represent a subset of tracked responses. (B) Test I experimental design. Experiment consisted of the delivery of pulse‐only trials (top trace) with a 50 ms long white noise pulse; prepulse + pulse trials consisting of prepulses at weak (second panel), medium (third panel) and high (bottom panel) intensities. Traces indicate the delivery of the auditory stimuli over time, with the prepulse delivery time corresponding to the starting time point. Red shading indicates the duration of stimulus delivery (50 ms each). Startle amplitudes from the startle window (orange shading) in pulse‐only trials were compared to the startle amplitudes from the startle windows in prepulse + pulse trials. (C) Test II experimental design. Two stimulus patterns were delivered namely a rhythmic and random pattern. For each, six white noise pulses of 50 ms each, onset indicated by dotted red line, duration indicated by the orange block, were delivered. Startle amplitudes in startle window 1 were compared to startle amplitudes in the subsequent five windows. Startle window duration is indicated in light orange. Observation windows indicated in gray were windows where a pulse was expected (dashed red line) but not presented.

Until recently, the scalability and potential clinical utility of detailed neurobehavioral assessments to measure these sensorimotor reflexes and behaviors have been limited by the need for specialized lab‐bound equipment (Cheng et al. 2018; Dwyer et al. 2023; Madsen et al. 2014). We have developed a user‐friendly smartphone‐based platform specifically designed for conducting neurobehavioral evaluations, called BlinkLab (Figure 1). BlinkLab is optimized for at‐home use, yielding robust results across various neurobehavioral tests and providing the opportunity to quantify sensorimotor functioning in large cohorts (Boele et al. 2023).

Here, we performed a case–control study to test, for the first time, whether BlinkLab smartphone‐based neurobehavioral assessments of sensorimotor function showed differences between children with autism and neurotypical children. We hypothesized that neurobehavioral measures including the ASR, PPI, and HAB would differ in autistic compared to neurotypical children. While analyzing these reflex‐based measures, we noticed distinct behaviors that more frequently occurred in children with autism compared to neurotypical children. Given the substantial body of evidence on differences in motor and sensorimotor behaviors in autism, we used our observations to guide our second, data‐driven aim where we compared sensorimotor behaviors in autistic and neurotypical children.

## 2 Methods and Materials

### 2.1 Participants

A cohort of 536 participants aged between 3 and 12 years was recruited (Table 1). Children diagnosed with autism, comprising 114 girls and 256 boys, were recruited at the Mohammed VI National Center for the Disabled (CNMH) at all eight locations in Morocco, including Fes, Salé, Safi, Marrakesh, Casablanca, Oujda, Tangier, and Agadir. These locations provided us with the opportunity to test unmedicated children in a population that is underrepresented in autism research (Durkin et al. 2015). The autism diagnosis was established prior to recruitment by a multidisciplinary team of specialists. The diagnostic procedure started with clinical observations based on symptoms observed and parent reports. Children were then referred to a pediatrician or neurologist to rule out any medical causes. Diagnoses are made according to the Diagnostic and Statistical Manual of Mental Disorders (American Psychiatric Association, D. S. M. T. F., and American Psychiatric Association 2013) criteria with different scales such as the Vineland Adaptive Behavior Scale used to evaluate different behavioral domains. Neurotypical controls, consisting of 72 girls and 94 boys without a neuropsychiatric diagnosis, were recruited from two schools, one in Taounate (90 km from Fes) and the other in Salé. All participants were selected regardless of sex, gender identity or race. Excluded were participants using medication that affects the nervous system (classified as ATC N0 medication, https://www.whocc.no), as such medication may modulate the acoustically evoked eyelid startle reflex (Geyer et al. 2001; Kumari et al. 2001). Written informed consent was obtained from the parents or caregivers of the children involved in the study, and verbal assent was given by the participating children, except in cases where children were non‐verbal. The study was performed in accordance with relevant guidelines and regulations and was reviewed and approved by the institutional review boards of Princeton University (#13943) and the Faculté de Médecine et de Pharmacie de Marrakech in Morocco (#23/2022).

**TABLE 1:** Demographic and clinical characteristics of participants.

Variable	All participants	Successful		Rejected	
		Autism	Neurotypical	Autism	Neurotypical
	*N* = 536	*N* = 275 (51.31%)	*N* = 156 (29.10%)	*N* = 95 (17.72%)	*N* = 10 (1.87%)
Girls	186 (34.70%)	79 (28.73%)	69 (44.23%)	35 (36.84%)	3 (30%)
Boys	350 (65.30%)	196 (71.27%)	87 (55.77%)	60 (63.16%)	7 (70%)
Age in years (mean ± SD)	7.51 (±2.10)	8.05 (±1.89)	6.97 (±2.11)	6.94 (±2.14)	6.20 (±2.61)
Age range (years)					
3–5	144 (26.87%)	47 (17.09%)	60 (38.46%)	32 (33.68%)	5 (50.00%)
6–8	241 (44.96%)	133 (48.36%)	68 (43.59%)	37 (38.95%)	3 (30.00%)
9–12	151 (28.17%)	95 (34.55%)	28 (17.95%)	26 (27.37%)	2 (20.00%)
Autism severity level					
Level 1	—	22 (8.00%)	—	—	—
Level 2	—	34 (12.36%)	—	—	—
Level 3	—	33 (12.00%)	—	—	—
Reason for rejection					
Volume	—	—	—	15 (15.79%)	1 (10.00%)
Headphone refusal/agitation	—	—	—	35 (36.84%)	3 (30.00%)
Premature test termination	—	—	—	45 (47.37%)	6 (60.00%)

Abbreviation: SD = standard deviation.

### 2.2 Experimental Setup

Neurobehavioral testing was performed using BlinkLab, a smartphone‐based platform (Boele et al. 2023) (Figure 1A). The tests included specific neurometric tests, including the ASR, PPI, and long‐term habituation (long‐term HAB) (Test I) and short‐term habituation (short‐term HAB) (Test II) along with general measurements of spontaneous and stimulus‐evoked postural, head, facial, and vocal responses (Tests I and II) (Abel et al. 1998; Dawson et al. 2018; Graham 1975; Krishnappa Babu et al. 2023; Martin et al. 2018; Swerdlow et al. 1999; Tenenbaum et al. 2020). Specifically, Test I (Figure 1B) consisted of pulse‐only trials as well as prepulse (of varying intensities from 5% to 25% of the intensity of the pulse) + pulse trials described in more detail in Appendix S1. Test II (Figure 1C) consisted of two habituation stimulus trains, namely a rhythmic (constant interval between stimuli) and random (inconsistent interval between stimuli) delivery of six white noise pulses. Children participated in these two 15‐min tests (Figures 1 and S1). For each trial, computer vision algorithms were used to track and record the position of the participant's facial landmarks over time.

During both tests, children viewed an audio‐compressed movie while auditory stimuli were presented. As highlighted by Blumenthal et al. (2006), background noise may influence measures such as PPI. To mitigate this, we carefully adjusted the background video audio track using the software package *Audacity* to ensure that the background sound pressure level, during playback on the phone's maximum volume setting, could not exceed a 65 dB sound pressure level. White noise auditory bursts were delivered through headphones. Audio compression and normalization were applied to maintain the signal within a controlled dynamic range, preventing distortion or clipping while ensuring a consistent background level. This controlled playback served as the auditory background for the white noise bursts presented during the smartphone‐based neurobehavioral tests. The measured sound levels during the movies were: minimum 23.8 dB, maximum 64.0 dB, LAF10 = 10% of the time > 45 dB, LAF50 = 50% of the time > 40 dB, and LAF90 = 90% of the time > 33 dB. To reduce the likelihood that children might fail to perceive the prepulses, as reported by Jones et al. (2015), we presented prepulses at three different intensities (Figure 1B), with the assumption that medium and high intensities would provide sufficient contrast relative to the background noise and the startle pulse.

### 2.3 Outcome Measures

For the analysis of the neurometric data, we used a similar data analysis pipeline to Boele et al. (2023), described in detail in Appendix S1. The following outcome measures were analyzed from Test I: the amplitude of acoustically evoked eyelid startle responses to pulse‐only trials (ASR) and prepulse + pulse trials (PPI), as well as the amplitude of the ASR over the course of the PPI experiment (long‐term HAB). From Test II, we analyzed the eyelid startle amplitude in response to the first pulse compared to the next five pulses for both the rhythmic and random stimulus patterns. We also analyzed the anticipatory eye blinks (AEB) in predefined observation windows (OW) containing an omitted pulse (Figure 1C). AEB were responses that were not triggered by the startle stimulus itself but rather by the expectation of an upcoming startle stimulus. The cumulative sum of the eyelid amplitude was also determined for the rhythmic and random HAB patterns.

Computer vision algorithms tracking and recording the position of the participants' facial landmarks over time allowed us to perform a data‐driven behavioral analysis where we began by listing behaviors that occurred more frequently in children with autism compared to neurotypical children. These behaviors included: general body movements, moving out of the phone camera view, head touches, headphone touches, pupil movements, mouth opening and vocalizations. We refined these behaviors to be clearly distinguishable and easily quantifiable. This resulted in 10 potential biomarkers, described in more detail in Appendix S1, where we quantified for Test I and Test II combined: (1) *Screen avoidance*: The percentage of trials wherein the child was not detectable in the smartphone's screen and camera. (2) *Headphone touches*: The percentage of trials wherein the child was touching the headphones. (3) *Vocalizations*: The percentage of trials wherein the child produced non‐syllabic vocalizations and the corresponding duration of occurrence. (4) *Anteroposterior postural stability*: The child's anteroposterior postural stability. (5) *Head rotations*: The percentage of trials wherein the child was rotating their head. (6) *Mouth openings*: The size and duration of the child's mouth openings and closings. (7) *Pupil range*: The mean horizontal pupil movement per session. (8) *Side eye*: How much a participant's pupil movement was in the opposite direction from the direction their head was turned. (9) *Baseline regression intercept*: The variation in baseline (pre‐stimulus) eyelid movements in the first five trials of a session. (10) *Baseline regression SD*: The stability of the variation in baseline eyelid movements across a session.

### 2.4 Statistical Analysis

Fisher's exact test was used to evaluate if there was a significant effect of diagnosis on the proportion of successful BlinkLab tests. To test for age differences between neurotypical and autistic children, we used the Wilcoxon rank sum test with continuity correction. Sex differences between the two diagnostic groups were tested using a chi‐square test. An ANOVA was used to test for age differences between children with autism with and without co‐occurring conditions.

Multilevel linear mixed‐effects models were used to assess the following group differences: eyelid startle amplitude for the different PPI stimulus types in Test I, eyelid amplitude of AEBs in Test II, and the maximum cumulative sum of eyelid amplitude in Test II. ANOVA on the mixed‐effect models was run for within group analyses of the effect of stimulus type on startle amplitude (PPI), the effect of trial number on startle amplitude (long‐term HAB) between and within groups, and the effect of pulse number and group on startle amplitude (short‐term HAB). Linear regression models were used to assess group differences in startle amplitude variability for the different PPI stimulus types, differences in Pearson correlation coefficients for regression of the ASR during PPI, and differences in the variability of the maximum cumulative sum of eyelid amplitude in a HAB experiment.

Multilevel binomial logistic regression was used to compare group differences in the trial‐level occurrence of the following behaviors: screen avoidance, headphone touches, and non‐syllabic vocalizations. For all other behaviors, linear regression models were used. Within each outcome category (e.g., eyelid closure amplitude in Test I), we corrected for multiple comparisons using the Bonferroni–Holm adjustment of *p*‐values.

For more experimental and statistical details, we refer to Appendix S1.

## 3 Results

We enrolled a total of 536 children between the ages of 3 and 12 years old, spanning the period between May 15, 2023, and December 7, 2024. BlinkLab tests were successfully administered in 431 (80.41%) children, of which 275 (63.81%) had autism and 156 (36.19%) were neurotypical (Table 1). Reasons for rejection are listed in Table 1. The proportion of successful tests differed significantly by diagnosis (*p* < 0.0001). The children with autism were significantly older than the neurotypical children (*W* = 14,472, *p* < 0.0001, *r* = −0.27, Table 1) and there was a significant difference in the proportion of males and females between the two groups (*χ* ^2^(1) = 10.61, *p* = 0.0011). To control for any possible effects of age or sex, we conducted all analyses with and without age and sex as confounders in all models and reported where age or sex had a significant effect. For 89 subjects (32.36%), we had information on the DSM‐V autism severity level, which is shown in Table 1. A summary of significant main diagnostic group effects is shown in Table 2 followed by the detailed analyses for each outcome variable. Effect sizes are reported as standardized *β* (where *β* values between 0.10 and 0.29 are considered small effects; 0.30–0.49 as moderate effects and 0.50 or greater considered large effects (Nieminen 2022)) for linear regression and LME models, and odds ratios are reported for multilevel binomial logistic regression models.

**TABLE 2:** Summary of outcome measures with significant main diagnostic group effects.

Outcome variable	Main effect diagnosis (*β*)	Effect of age	Effect of sex	Effect of co‐occurring condition
Test I (Figure 2)				
Pearson correlation coefficient for regression of ASR pulse‐only trials	−0.13	No	No	—
pp10% + pulse trials eyelid closure amplitude	0.20	No	No	No
pp25% + pulse trials eyelid closure amplitude	0.27	No	No	Yes
Pulse‐only trials eyelid closure amplitude variability	0.19	No	No	No
pp5% + pulse trials eyelid closure amplitude variability	0.17	No	No	No
pp10% + pulse trials eyelid closure amplitude variability	0.24	No	No	No
pp25% + pulse trials eyelid closure amplitude variability	0.19	No	No	No
Test II (Figure 3C,D)				
Rhythmic trials cumulative sum of eyelid closure amplitude	0.18	No	No	No
Random trials cumulative sum of eyelid closure amplitude	0.18	Yes	No	No
Rhythmic trials variability in the maximum cumulative sum of eyelid closure amplitude	0.34	Yes	No	No
Random trials variability in the maximum cumulative sum of the eyelid closure amplitude	0.28	No	No	No
Test I and II (Figure 4)				
Screen avoidance	6.09^a^	Yes	No	No
Headphone touches	16.67^a^	No	No	No
Non‐syllabic vocalizations	95.33^a^	No	No	No
Anteroposterior postural stability	0.48	No	No	No
Head rotations	0.54	No	No	No
Mouth openings	0.55	No	No	Yes
Pupil range	0.47	No	No	No
Side eye	0.27	No	No	No
Baseline regression intercept	0.36	No	No	No
Baseline regression SD	0.4	No	No	No

Abbreviations: SD = standard deviation; pp = prepulse.

^a^ Odds ratio reported in place of *β* as multilevel binomial logistic regression models were used for these outcome measures.

### 3.1 Acoustic Startle Response

**FIGURE 2:**
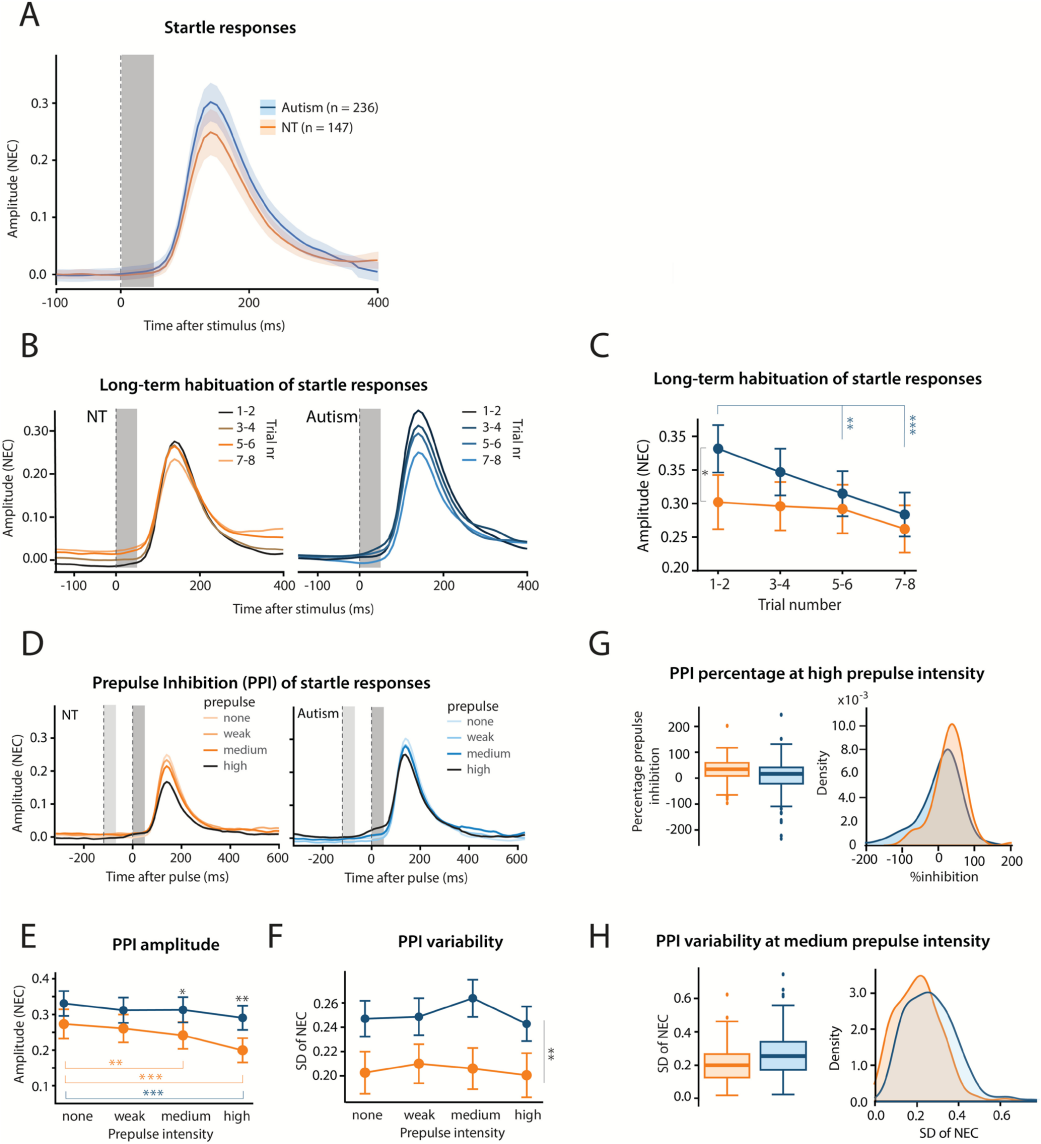
Characteristics of the auditory startle response, long‐term startle habituation, and prepulse inhibition in children with autism and neurotypical (NT) children. (A) Group averaged eyelid movement plots showing the acoustically evoked startle responses for children with autism (blue) and NT children (orange). At a group level, children with autism and NT children had comparable eyelid startle responses, however, when sex was accounted for, children with autism had significantly higher startle responses than NT children. (B) Group averaged eyelid movement plots of the pulse‐only trials ordered by trial number. Trials 1–2 (dark coloring) were delivered at the start and trials 7–8 (light coloring) at the end of the 15‐min Test I. (C) Mean eyelid startle amplitude as a function of trial number for children with autism and NT children. Children with autism showed significant long‐term HAB whereas NT children did not. (D) Group averaged eyelid movement plots ordered by stimulus type, that is, by the intensity of the prepulse that preceded the pulse. For panels A, B and D, the left vertical dashed line indicates the onset of the auditory prepulse, the right one indicates the onset of the pulse. Gray vertical columns indicate the stimulus durations of 50 ms. (E) Mean amplitude of the eyelid startle response as a function of prepulse intensity. The group with NT children exhibited a typical prepulse inhibition (PPI) response, characterized by a decrease in mean eyelid startle amplitude as the intensity of the prepulse increased. The group of children diagnosed with autism showed some evidence of PPI, however, only for the highest prepulse intensity trials. (F) Variability of the amplitude of the eyelid startle responses as a function of prepulse intensity for children with autism and NT children. For all prepulse intensities the group of children with autism had a higher variability than NT children. (G) Box and density plots showing the percentage of PPI in trials with a high (25% of pulse) prepulse intensity. Subjects with a median startle amplitude for pulse trials < 0.075 were classified as non‐responders and excluded from this plot. In more than 50% of the trials there was no decrease, but even an increase, in the startle response amplitude in children with autism. (H) Increased variability in the amplitude of the eyelid startle response during trials with a medium prepulse intensity in children with autism compared to NT children. Colored shading in panel A and error bars in panels C, E, F indicate 95% confidence intervals. Significance levels: * *p* < 0.05, **p < 0.01, ***p < 0.001. All data and statistical analyses are available in Tables S1 and S2. Figure S3 shows typical examples of raw eyelid movement profiles during PPI.

We did not detect a statistically significant difference in the startle amplitude in the pulse‐only trials between autistic and neurotypical children (*b* = 0.05, *t*_381_ = 1.82, CI [−0.004 to 0.09], *p* = 0.070; Figure 2A; Table S1A). The mean eyelid startle amplitude for children with autism was 0.33 (±0.34), while in neurotypical children it was 0.29 (±0.31). Sex was a significant confounder (*b* = −0.06, *t*_380_ = −2.52, CI [−0.11 to −0.01], *p* = 0.012; Table S1A); however, age was not. In the model including sex, diagnosis did have a significant effect on the startle amplitude (*b* = 0.05, *t*_380_ = 2.19, CI [0.01–0.10], *p* = 0.029).

### 3.2 Long‐Term Habituation

Over the course of the 15‐min Test I, children with autism showed significant habituation. There was a significant decrease (*b* = −0.03, CI [−0.04 to −0.02]) in startle amplitude from 0.38 (±0.36) in the first two trials to 0.28 (±0.32) in trials 7–8 (Figure 2B,C). In neurotypical children, while there was a decrease in startle amplitude from 0.30 (±0.34) in trials 1–2 compared to 0.26 (±0.29) in trials 7–8, this decrease was not significant (*b* = −0.01, CI [−0.02 to 0.003]; Table S2A). While there was no main effect of diagnosis (*F*_1,381_ = 3.28, *p* = 0.071), the main effect of trial number (*F*_1,2237_ = 26.63, *p* < 0.0001) and trial number × diagnosis interaction effects (*F*_3,2237_ = 5.59, *p* = 0.018) were significant. Sex was a significant confounder (*F*_1,380_ = 6.48, *p* = 0.011); however, age was not (Table S2A). The significant decrease in startle amplitude over the course of Test I in autistic but not NT children is further supported by the linear regression analysis of ASR amplitudes (Figure S2; Table S2B). The mean Pearson correlation coefficient for children with autism was −0.16 (±0.47), in comparison to −0.04 (±0.42) for NT children. There was a significant effect of diagnosis on the Pearson correlation coefficient (*b* = −0.12, *t*_381_ = −2.53, CI [−0.21 to −0.03], *p* = 0.048). This was only the case for pulse‐only trials. No significant correlation was found for children with autism or neurotypical children for any of the prepulse categories (Figure S2; Table S2B).

### 3.3 Prepulse Inhibition

Children with autism demonstrated reduced levels of PPI and increased variability in their startle eyelid responses (Figures 2D–H and S3 and Table S1A,B). In neurotypical children, startle amplitude decreased with increasing prepulse intensities from 0.29 (±0.31) in trials without a prepulse to 0.25 (±0.30) in trials with a prepulse intensity at 10% of the pulse intensity (*t*_4164_ = 3.64, *p* = 0.0006) and 0.22 (±0.28) in trials with the maximum prepulse intensity (*t*_4164_ = 7.49, *p* < 0.0001). In contrast, children with autism did not exhibit a corresponding amplitude reduction (*t*_5868_ = 1.74, *p* = 0.16) from pulse‐only trials (mean = 0.33 ± 0.34) to prepulse 10% trials (mean = 0.32 ± 0.35). However, they did show a significant decrease from the pulse trials to a mean of 0.30 (±0.34) for trials with the maximum prepulse intensity (*t*_5868_ = 4.02, *p* = 0.0003). We found a statistically significant effect of diagnosis for the trials with prepulse intensities at 10% (*b* = 0.07, *t*_380_ = 2.82, CI [0.02–0.11], *p* = 0.010) and 25% (*b* = 0.09, *t*_376_ = 3.73, CI [0.04–0.13], *p* = 0.001). Reported *p*‐values were Bonferroni–Holm adjusted for three comparisons.

There were no significant confounding effects of age or sex for any of the prepulse intensities, nor a significant interaction effect of age × diagnosis. Interestingly, children with autism exhibited significantly increased variability in response amplitudes for all prepulse intensities and neither sex nor age was a significant confounder (Figure 2F,H; Table S1A).

### 3.4 Short‐Term Habituation

**FIGURE 3:**
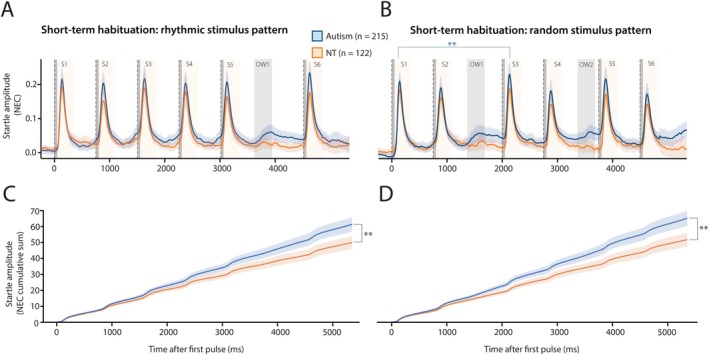
Short‐term startle habituation and anticipatory eyeblinks in a rhythmic and random stimulus pattern (A, B) Group averaged plots of eyelid movement over a duration of five seconds during which eyelid startle responses were evoked by the presentation of six white noise pulses in either a rhythmic (A) or a more random (B) stimulus pattern. Each 15‐min smartphone experiment consisted of ten rhythmic and ten random trials. Onset of the white noise pulses is indicated with the vertical dashed lines, duration of pulses (50 ms each) by the gray vertical columns. The magnitude of the startle pulse‐evoked eyelid movements, expressed as normalized eyelid closure (NEC), was quantified in the ‘startle windows’ indicated by the yellow shaded areas S1–S6. The magnitude of anticipatory eyeblink (AEB) responses around the moment of omitted stimuli was quantified in the gray‐shaded observation windows (OW) 1 and 2. NT children showed no short‐term habituation and children with autism even showed sensitization (i.e., startle responses becoming larger after the first pulse) in the random stimulus pattern. (C, D) Group averaged plots showing the cumulative sum of eyelid movements shown in panels A and B. The cumulative sum was higher and more variable in children with autism compared to NT children during the rhythmic and random trials. Colored shading indicates 95% confidence intervals. Significance levels: * *p* < 0.05, **p < 0.01, ***p < 0.001. All data and statistical analyses are available in Table S3. Figure S4 shows typical examples of raw eyelid movement profiles during short‐term HAB.

In both the rhythmic and random stimulus patterns, we observed no decrease (i.e., habituation) in the mean amplitude of eyelid startle responses over the course of the six startle pulses. This lack of habituation was consistent across both children with autism and neurotypical children and neither sex nor age were significant confounders (Figures 3A,B and S4; Table S3A). Notably, reflexive startle amplitudes tended to increase slightly after the first pulse in the group with autism, with a statistically significant startle amplitude increase at pulse 3 in the random stimulus pattern (pulse 1 vs. pulse 3: *t*_16767_ = −3.19, *p* = 0.0070).

### 3.5 Anticipatory Eye Blinks

Here we looked at whether eye blinks were present during windows where stimuli were omitted in Test II. We did not find statistically significant differences in eyelid closure amplitude in any of the predefined observation windows for either the rhythmic or random pulse trains (Table S3B). While sex was not a significant confounder, age was for observation window 1 in the rhythmic protocol (*b* = 0.01, *t*_332_ = 2.32, CI [0.002–0.02], *p* = 0.021) and random protocol (*b* = 0.01, *t*_334_ = 2.19, CI [0.001–0.02], *p* = 0.029) as well as for observation window 2 in the random protocol (*b* = 0.01, *t*_334_ = 3.46, CI [0.01–0.02], *p* = 0.0006). When quantifying short‐term HAB in conjunction with AEBs, using the cumulative summation of the mean eyelid amplitude in the two short‐term HAB protocols, we did see differences between children with autism and neurotypical children. The cumulative sum for the rhythmic protocol was significantly different (*b* = 18.06, *t*_333_ = 2.94, CI [5.97–30.15], *p* = 0.0035; Figure 3C; Table S3C) between autistic children (cumulative eyelid closure across 10 trials = 78.36 ± 108.98) and neurotypical children (cumulative eyelid closure across 10 trials = 61.00 ± 75.42). For the random stimulus pattern, the mean cumulative sum of the eyelid amplitude was significantly higher (*b* = 19.16, *t*_335_ = 2.99, CI [6.54–31.78], *p* = 0.0030; Figure 3D; Table S3C) in children with autism (cumulative eyelid closure = 81.90 ± 114.52), compared to typically developing children (cumulative eyelid closure = 63.92 ± 83.17). Age was a significant confounder (*b* = 3.73, *t*_334_ = 2.43, CI [0.71–6.75], *p* = 0.016); however, the main effects of autism stayed significant in the model adjusted for age (*b* = 15.48, *t*_334_ = 2.37, CI [2.62–28.35]; *p* = 0.019; Table S3C). We also quantified the variability in the maximum cumulative sum of the eyelid closure amplitude for the rhythmic and random stimulus patterns (Table S3C). Children with autism showed significantly more variability (*b* = 32.91, *t*_335_ = 6.60, CI [23.10–42.73], *p* < 0.0001) in the maximum cumulative sum of eyelid closure amplitude (mean SD = 87.14 ± 48.96) compared to neurotypical children (mean SD = 54.22 ± 33.52) in the rhythmic stimulus pattern. The same was true for the random stimulus pattern (*b* = 28.91, *t*_335_ = 5.36, CI [18.30–39.52], *p* < 0.0001) where autistic children had a mean SD of 89.55 (±52.52) and neurotypical children had a mean SD of 60.64 (±37.30). For the rhythmic pattern, age was a significant confounder (*b* = 2.38, *t*_335_ = 2.00, CI [0.04–4.73], *p* = 0.047); however, the effect of diagnosis remained significant (*b* = 30.61, *t*_335_ = 6.00, CI [20.58–40.64], *p* < 0.0001).

All *p*‐values reported below were adjusted for 10 tests using the Bonferroni–Holm method.

### 3.6 Screen Avoidance

**FIGURE 4:**
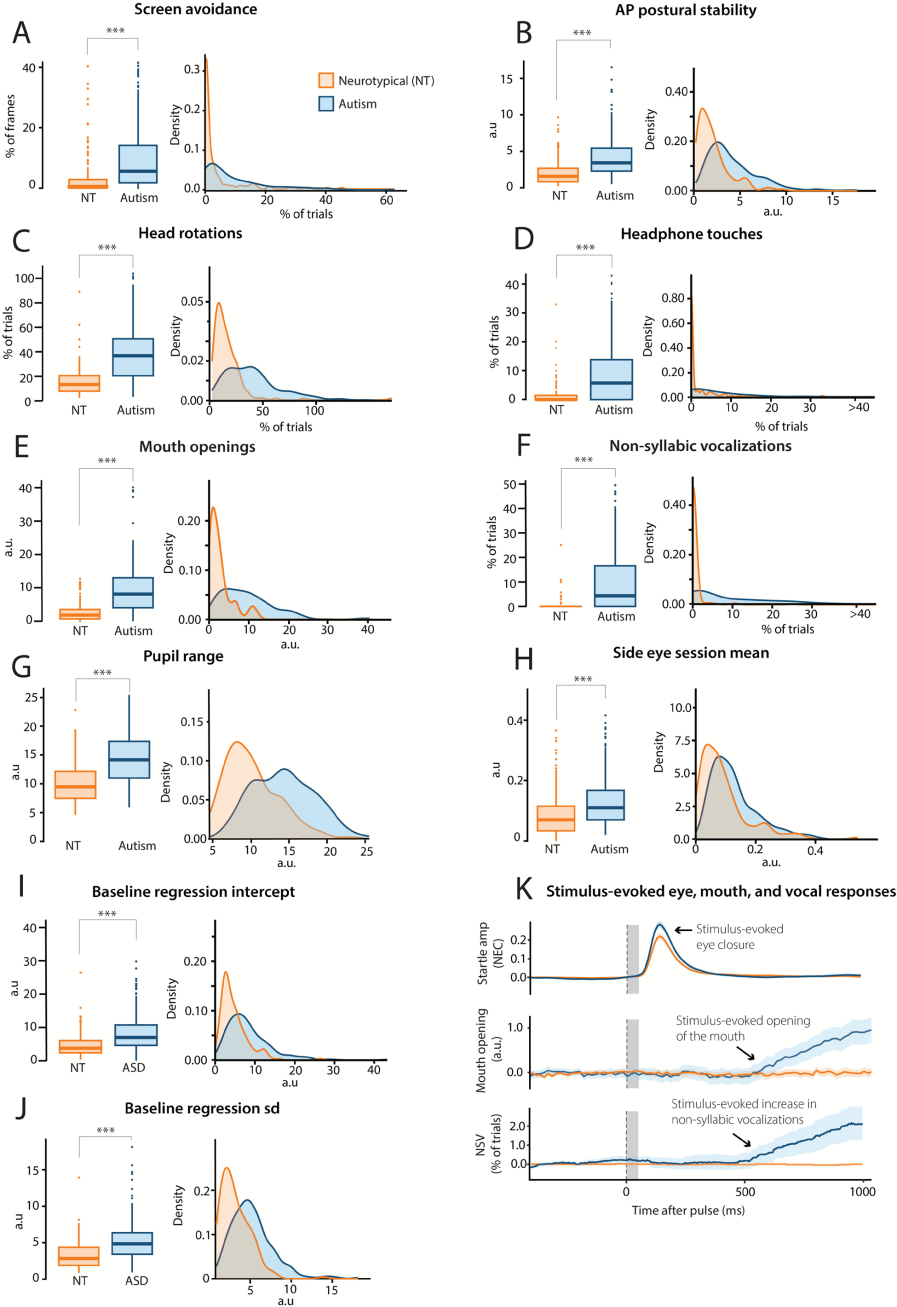
Postural, head, facial, vocal and eyelid responses during the smartphone neurobehavioral evaluations differ between children with autism and neurotypical (NT) children. (A–J) Box plots and density plots illustrate increased response levels in children with autism (blue) compared to NT children (orange). A missing cap at the whiskers of the box plot means that the whiskers are not capturing the full data range, the range in density plots can therefore extend the range in the box plot. (K) Group averaged profiles of startle eyeblinks (normalized eyelid closure, NEC), and baseline‐corrected mouth openings and non‐syllabic vocalizations (NSV) around the onset of the auditory stimulus. Vertical dashed line indicates the onset of the auditory pulse. Gray vertical columns indicate the stimulus durations of 50 ms. The auditory stimulus evokes a startle eyeblink with a short latency to onset, followed by a mouth opening and vocalization with a longer latency to onset. Blue and orange shadings indicate the 95% confidence intervals. Significance levels: * *p* < 0.05, **p < 0.01, ***p < 0.001. All data and statistical analyses are available in Table S4. AP = anterior posterior; SD = standard deviation.

Autistic children avoided watching the smartphone's screen significantly more than neurotypical children (odds ratio (OR) = 6.09, CI [4.16–8.93], *p* < 0.0001; Figure 4A; Table S4). On average, children with autism displayed this behavior in 9.94% (±11.56) of trials, while neurotypical children faced away in 3.66% (±7.76) of trials. Age was a significant confounder (OR = 0.84, CI [0.77–0.92], *p* = 0.001); however, the main effects of autism remained significant in the model adjusted for age (OR = 7.26, CI [4.91–10.75], *p* < 0.0001).

### 3.7 Anteroposterior Postural Stability

Children with autism exhibited significantly more anteroposterior postural movements during the experiments compared to their neurotypical counterparts (*β* = 0.48, CI [0.83–1.17], *p* < 0.0001; Figure 4B; Table S4). Children with autism demonstrated a mean standard deviation of the facial bounding box size of 4.24 (±2.82), whereas neurotypical children exhibited a value of 2.11 (±1.77).

### 3.8 Head Rotations

Autistic children demonstrated significantly more head rotations compared to neurotypical children (*β* = 0.54, CI [0.95–1.28], *p* < 0.0001; Figures 4C and S4). Children with autism exhibited head rotations in 40.22% (±25.62) of trials while neurotypical children displayed head rotations in 16.62% (±16.76) of trials.

### 3.9 Headphone Touches

Children with autism displayed significantly more headphone touches during the experiments (OR = 16.67, CI [10.28–27.04], *p* < 0.0001; Figure 4D; Table S4). Children with autism displayed headphone touches in 11.31% (±16.74) of trials, contrasting with neurotypical children who exhibited this behavior in 1.87% (±5.97) of trials.

### 3.10 Mouth Openings

Autistic children displayed both an increased frequency and larger mouth openings compared to neurotypical children (*β* = 0.55, CI [0.97–1.30], *p* < 0.0001; Figure 4E,K; Table S4). The mean mouth surface area for autistic children was 9.55 (±6.95) versus a mean of 2.96 (±3.02) in neurotypical children. In addition, we were able to elicit mouth opening with auditory stimuli in children with autism, a response not observed in neurotypical controls (Figure 4K).

### 3.11 Vocalizations

Children with autism demonstrated a significantly higher percentage of non‐syllabic vocalizations during the experiments (OR = 95.33, CI [45.44–199.97], *p* < 0.0001; Figure 4F; Table S4). Notably, these vocalizations occurred spontaneously throughout the 15‐min experiments in addition to being elicited by auditory stimuli. As with mouth openings, these stimulus‐elicited responses were not observed in neurotypical controls (Figure 4K). Non‐syllabic vocalizations were observed in 11.16% (±15.21) of trials in autistic children, in contrast to 0.43% (±2.40) of trials in neurotypical children (Figure 4F).

### 3.12 Pupil Range

Autistic children displayed significantly more horizontal pupil movements compared to neurotypical children (*β* = 0.47, CI [3.45–4.97], *p* < 0.0001; Figure 4G; Table S4). In autistic children, the mean horizontal pupil movement was 14.33 (±4.04) versus a mean of 10.12 (±3.48) in neurotypical children.

### 3.13 Side Eye

Children with autism also tended to more frequently look in the opposite direction from where their head was turned, referred to in this study as “side‐eyeing” (*β* = 0.27, CI [0.36–0.74], *p* < 0.0001; Figure 4H; Table S4). The mean amount of side‐eye in children with autism was 0.13 (±0.08) compared to 0.09 (±0.08) in neurotypical children.

### 3.14 Baseline Regression Measures

More variation in baseline eyelid opening was observed in autistic children. Children with autism showed more variation in baseline eyelid opening in the early stages of the experiment (quantified by the baseline regression intercept; *β* = 0.36, CI [0.57–0.94], *p* < 0.0001; Figure 4I; Table S4). The mean baseline regression intercept was 8.31 (±5.55) in autistic children compared to 4.77 (±3.51) in neurotypical children. This variation in baseline eyelid opening was also more variable in children with autism compared to neurotypical children (*β* = 0.40, CI [0.65–1.02], *p* < 0.0001; Figure 4J; Table S4), indicated by a higher baseline regression SD in autistic (mean = 5.23 (±2.60)) compared to neurotypical children (mean = 3.27 (±1.85)).

### 3.15 Exploratory Analyses

To explore whether the outcomes described above were indeed autism‐related and not due to other diagnoses that frequently co‐occur with autism, we performed sub‐analyses for the effect of co‐occurring conditions on outcome measures that differed significantly between neurotypical and autistic children. For these analyses we focused on the two most common co‐occurring conditions in this sample, which were intellectual disability (ID) and attention‐deficit hyperactivity disorder (ADHD) (Khachadourian et al. 2023). Information on co‐occurring conditions was available for 92% of the children with autism (Table S5). While there was a significant effect of the co‐occurring conditions group on age (*F*_3,249_ = 2.89, *p* = 0.036), none of the three co‐occurring condition groups differed significantly in age from the group without co‐occurring conditions (ADHD vs. autism only: *t*_249_ = −1.30, *p* = 0.196, ID vs. autism only: *t*_249_ = 1.94, *p* = 0.053, ADHD & ID vs. autism only: *t*_249_ = −0.46, *p* = 0.650).

The only co‐occurring condition group to show a significant difference in the amplitude of the ASR compared to the group without co‐occurring conditions was the group with both ADHD and ID (*b* = −0.10, *t*_214_ = −2.15, CI [−0.19 to −0.01], *p* = 0.033, Table S6A). There were no significant effects of co‐occurring conditions on long‐term HAB (Table S7).

In the prepulse trials with the prepulse intensity at 25% of the pulse, children with autism and ID showed a significantly smaller startle amplitude compared to children with autism and no co‐occurring conditions (*b* = −0.12, *t*_209_ = −3.04, CI [−0.19 to −0.04], *p* = 0.008, Table S6A). Of the groups of children with autism, the group with ADHD only (*F*_3,717_ = 2.66, *p* = 0.047) and ID only (*F*_3,1623_ = 8.73, *p* < 0.0001) showed a significant effect of the prepulse on eyelid closure amplitude, whereas children with both ADHD and ID and those without co‐occurring conditions did not show significant PPI (Table S6B).

There was no effect of co‐occurring conditions on short‐term habituation for both the rhythmic and random stimulus patterns (Table S8A,B). Similarly, there was no effect of the co‐occurring conditions on the cumulative sum of the mean eyelid amplitude or the variability of the maximum cumulative sum in either of the two short‐term HAB protocols (Table S9).

Mouth opening was the only sensorimotor behavior where we found a statistically significant effect of co‐occurring condition in the children with autism. Specifically, autistic children with both ADHD and ID showed significantly more mouth openings (mean = 12.66 ± 8.13) compared to children with autism and no co‐occurring conditions (mean = 8.40 ± 5.60, *β* = 0.59, CI [0.24–0.85], *p* = 0.0056, Table S10).

## 4 Discussion

In this study we assessed the ability of smartphone‐based neurobehavioral quantification of sensorimotor functioning to distinguish between children with and without autism. We successfully evaluated 431 children aged 3–12 years with the BlinkLab smartphone platform. In this large sample, we confirmed that autistic children showed diminished PPI levels compared to neurotypical children. When accounted for sex, which was a significant confounder, the amplitude of the ASR was higher in autistic compared to neurotypical children. Interestingly, children with autism showed significantly stronger long‐term habituation of the ASR compared to neurotypical children. Furthermore, children with autism showed more variability in eyelid closure in both Test I (ASR, PPI, long‐term HAB) and Test II (short‐term HAB), which was not better explained by age, sex or the presence of co‐occurring conditions. In addition, several of the spontaneous and evoked behavioral parameters acquired with our digital platform showed significant differences amongst the diagnostic groups.

### 4.1 Reduced Sensorimotor Gating in Children With Autism

The current study is one of the first to exploit smartphone technology and computer vision analysis to study simple forms of sensorimotor behavior in autism, including PPI and habituation of acoustically evoked eyelid startle responses. Similar to studies done with smaller numbers of subjects in a laboratory setting (Cheng et al. 2018; Dwyer et al. 2023; Madsen et al. 2014; McAlonan et al. 2002; Perry et al. 2007), we found lower levels of PPI in autism in terms of eyelid closure amplitude. Since PPI is considered a measure for sensorimotor gating (Braff et al. 2001; Swerdlow et al. 1999), this finding corresponds to the atypical sensorimotor processing patterns commonly reported in autism (Kanner 1943; Kern et al. 2006; Marco et al. 2011; Mosconi and Sweeney 2015). Interestingly, our exploratory analyses suggest that PPI in autism may be affected by the presence of co‐occurring conditions. Children with autism and intellectual disability showed significantly lower eyelid closure amplitudes at the strongest prepulse intensity compared to children with autism only. There is a lack of consensus on how co‐occurring conditions like intellectual disability affect sensory processing in autism (Werkman et al. 2023), and future studies should further examine their potential impact on PPI in autism.

### 4.2 Stronger Long‐Term Habituation in Autistic Children

Interestingly, children with autism did show significant habituation over a longer time period, that is, over the course of the 15 min Test I. The divergence in the eyelid startle amplitude between neurotypical children and children with autism is significant for early trials and then decreases over the course of subsequent trials, which indicates that the startle amplitude in early trials possibly drives the observed difference in habituation.

### 4.3 No Short‐Term Habituation with Sub‐Second Interstimulus Intervals, but Autism‐Specific Sensitization

By using both rhythmic and random stimulus trains, we also determined the level of short‐term habituation (Test II). To our surprise, neither neurotypical nor children with autism showed decrements in their responses following repeated stimulation with a train of white noise pulses. In contrast, in a previous study (Boele et al. 2023), we found that a community sample of young adults does show short‐term startle habituation to a similar stimulus pattern. Our finding does coincide though with a lab‐based study (Muenssinger et al. 2013), which found weak or even no habituation in children exposed to blocks of eight tones separated by 300 ms. Importantly, in both this study and that of Muenssinger et al. (2013), we evaluated habituation to identical stimuli presented in a train with sub‐second time intervals between stimuli. Muenssinger et al. (2013) proposed that children perceiving the individual stimuli but processing them as one stimulus may explain the lack of habituation within a stimulus train. In contrast, in Test II, autistic children, unlike neurotypical children, showed a slight increase in their startle amplitudes after the first 2 pulses in the random stimulus train, corresponding with previous findings (Madsen et al. 2014) and potentially reflecting stronger sensitization.

### 4.4 Increased Startle Response Variability in Children with Autism

In addition, we found that autistic children had increased variability in their eyelid startle response amplitudes in both Tests I and II. This is consistent with the frequently reported increased inter‐individual variability in autism not only at a behavioral level (Haigh 2018; Haigh et al. 2016), but also at a neural level (Bhaskaran et al. 2023; Haigh 2018; Milne 2011). Likewise, autistic children showed both a higher cumulative sum of eyelid amplitude and variability in the maximum cumulative sum of eyelid amplitude in Test II, suggesting not merely a hyper‐responsiveness to repeated auditory stimuli, but also more inconsistencies in this hyper‐responsiveness. Together, our data raise the possibility that variability in sensory motor processing rather than a simple hypo‐ or hyper‐responsiveness to sensory stimuli may better explain autism‐related sensory differences.

### 4.5 Different Behavioral Responses in Autistic Children

Together with the above‐described differences in neurometric measures, we also found clear differences in the behavioral responses during both Tests I and II between autistic and neurotypical children. Specifically, non‐syllabic vocalizations were more common in autistic children, aligning with prior research (Tenenbaum et al. 2020). Likewise, postural movements and mouth openings were increased in children with autism, as well as head rotations, headphone touches, and screen avoidance. These results are compatible with other studies using video presentations (Dawson et al. 2018; Krishnappa Babu et al. 2023; Martin et al. 2018). It is unlikely that these behaviors were driven by conditions that frequently occur with autism, as all but the mouth openings showed no differences between autistic children with and without co‐occurring conditions.

### 4.6 Study Strengths and Limitations

Strengths of our study lie in evaluating the BlinkLab app in an everyday environment with a sample of unmedicated children identified through a multi‐center design. Existing technologies for autism often conduct research and validation studies in predominantly white, Western, male populations with direct access to optimal medical care (Ponzo et al. 2023). By conducting our study in a more sex‐balanced and geographically underrepresented sample, we aim to reduce existing barriers and enhance the technology's applicability on a global scale (Dawson et al. 2023; Kirby et al. 2022).

Smartphone‐mediated neurobehavioral assessments present novel technical challenges, particularly when assessing young children. These challenges include issues such as experimental disruptions caused by unreliable Wi‐Fi networks, accidental or deliberate disconnection of headphones or adjustments to the volume settings, improper positioning of the smartphone, and instances where the child covers the selfie camera. These issues are potentially addressable with extensions to the software platform's user interface and feedback mechanisms. Substantial effort was also devoted to making these procedures both feasible and engaging for young children. This required pragmatic adjustments, including the use of a short entertaining video with sound, to sustain attention during the 15 min of testing. While such measures constitute minor compromises in experimental rigor (Blumenthal et al. 2006; Jones et al. 2015), they were essential for successfully testing a large cohort of young neurotypical and autistic children.

This study was limited by the lack of detailed clinical characterization of the participants and the over‐representation of children with autism compared to the global prevalence of the disorder. Future work should ensure neurotypical children are also evaluated by medical professionals and could investigate potential correlations between symptom severity and smartphone neurobehavioral measures. Stratifying children by level of functioning was, however, beyond the scope of this study where the primary aim was to first assess whether smartphone‐based neurobehavioral measures could distinguish differences in sensorimotor functioning. Another limitation was that children were not formally screened for visual and auditory abnormalities. If a significant group of children with these problems were present in our sample some effects may have been underestimated.

Expanding this retrospective case–control study to a pre‐registered prospective clinical trial would be a next step for future work to evaluate the potential diagnostic accuracy of a smartphone tool for autism. Prior to this, it would also be important to better characterize the sample of children with autism that are unable to complete the BlinkLab tests and further improve the tests for these children. Finally, repeated measurements from a cohort will allow the effects of behavioral and pharmacological interventions on sensorimotor function to be quantified longitudinally.

## 5 Conclusion

Our smartphone‐based testing allows the objective assessment of sensorimotor processing in children with autism, an important yet neglected topic despite its significant impact on the quality of life of individuals with autism (Narzisi et al. 2025). We have revealed highly significant general sensorimotor behavioral differences, such as vocalizations, head‐ and postural movements, between neurotypical children and those with autism. While these general behavioral measures lack the specificity of neurobehavioral reflex‐based measures, combining them with neurometrics such as ASR, PPI, and HAB measures may hold the best potential in differentiating between individuals with and without autism. Since real‐world smartphone‐based tests do not require verbal or social interaction, our study may close the research gaps arising from the historical exclusion of non‐verbal individuals with autism from research, although a better understanding of those unable to complete the BlinkLab tests is needed. In addition, our study complements other testing platforms that measure social interactions using human scoring of behavior. Our results serve as a starting point from which to investigate the diagnostic accuracy of accessible technology in future prospective clinical trials.

## Author Contributions

H.‐J.B., S.S.‐H.W., A.U., C.P.B., S.K.E.K. conceived and designed the study. H.‐J.B., S.S., A.E.I., S.S.‐H.W., A.U., C.P.B., S.K.E.K., K.D.G., L.E.M.R., S.L. were responsible for the methodology and H.‐J.B., S.S.‐H.W., A.E.I., L.E.M.R., S.K.E.K., K.D.G., S.M., T.F.S., V.C.‐A., Y.A. carried out the investigation. The analysis was conducted by H.‐J.B., C.P.B., S.K.E.K., S.S., K.D.G., S.L., M.J.O. and visualization was done by H.‐J.B., S.K.E.K., and K.D.G. H.‐J.B. and S.S.‐H.W. acquired funding for this project. Project administration was carried out by H.‐J.B., S.S., A.E.I., V.C.‐A., L.E.M.R., K.D.G., K.B., S.A.B., Y.A. H.‐J.B., A.U., S.K.E.K., C.I.D.Z., S.S.‐H.W., M.J.O. were responsible for supervision. The original draft of the manuscript was written by K.D.G., H.‐J.B., M.J.O. and reviewed and edited by H.‐J.B., K.D.G., M.J.O., S.L., C.J., S.S., C.P.B., C.I.D.Z., S.S.‐H.W., A.E.I., L.E.M.R., E.S., Y.A. All authors had the final responsibility for the decision to submit for publication.

## Funding

This work was financially supported by the Princeton University Accelerator Grant and BlinkLab Limited.

## Ethics Statement

The study was performed in accordance with relevant guidelines and regulations and was reviewed and approved by the institutional review boards of Princeton University (#13943) and the Faculté de Médecine et de Pharmacie de Marrakech in Morocco (#23/2022).

## Consent

The researcher and parents of the children pictured provided informed consent for publication of these images in an online open‐access publication.

## Conflicts of Interest

H.‐J.B., S.K.E.K., C.P.B., S.S.‐H.W., A.U., and C.I.D.Z. engage with BlinkLab Limited as licensors of the technology, as co‐founders, and as equity holders. S.S., C.P.B., L.E.M.R., K.D.G., S.L., M.J.O., T.F.S. are employed by BlinkLab Limited. The remaining authors declare no competing interests.

## Supporting information


**APPENDIX S1:** Supporting Information.

## Data Availability

The datasets generated and analyzed during the study are available on: https://github.com/BlinkLab‐Ltd/ASD‐dataset.
